# Deep convolutional neural network based archimedes optimization algorithm for heart disease prediction based on secured IoT enabled health care monitoring system

**DOI:** 10.1038/s41598-025-12581-8

**Published:** 2025-07-25

**Authors:** Sureshkumar S, Santhosh Babu A. V, Joseph James S, Maranco M

**Affiliations:** 1 P. A. College of Engineering and Technology, Pollachi, 642002 India; 2 Information Technology, Hindusthan Institute of Technology, Coimbatore, 641032 India; 3 Computational Intelligence, College of Engineering and Technology, SRM Institute of Science and Technology, Kattankulathur, Chennai, 603203 India; 4 Networking and Communications, College of Engineering and Technology, SRM Institute of Science and Technology, Kattankulathur, Chennai, 603203 India

**Keywords:** Heart disease prediction, IoT-based healthcare monitoring system, Archimedes optimization algorithm, DCNN, Deep learning, Medical data security, Matrix-based RSA encryption, Secure health monitoring, Biomedical signal processing, Intelligent health diagnostics, Biomedical engineering, Health care, Health services

## Abstract

The Internet of Things (IoT) is a rapidly evolving and user-friendly technology that connects everything and enables effective communication between linked things. In hospitals and other healthcare centers, healthcare monitoring systems have exploded in popularity over the last decade, and wireless healthcare monitoring devices using diverse technologies have a huge interest in several countries worldwide. The existing studies in healthcare IoT met a few shortcomings in terms of privacy, security, higher data dimensionality, higher cost, larger execution time, and so on. To tackle these issues, we proposed a novel IoT-enabled and secured healthcare monitoring framework (IoT-SHMF) for heart disease prediction. The data are taken from the Cleveland Heart Disease database. First, authentication is performed through registration, login, and patient data verification. The Matrix-based RSA encryption technology and a blockchain-based data storage concept provide safe data transmission and authorization. Subsequently, the secured data is downloaded by the hospital management (HM) system. The HM system scrutinizes the decrypted data. Finally, the Deep Convolutional Neural Network-based Archimedes Optimization (DCNN-AO) algorithm classifies the normal and abnormal classes of heart disease. The implementation work of the proposed model is simulated using JAVA software with different performance measures. Various performance metrics with state-of-art methods validate the effectiveness of the proposed model. The proposed IoT-based system ensures better security by about 98%. The decryption time of our proposed approach, when the sensor nodes are equal to 25, is 37 seconds.

## Introduction

Major attention is paid to health and healthcare systems adopting a model in every society. The overcrowding and lack of basic services in rural communities create several health issues and skin disorders^1^. Because of the tremendous population increase, conventional healthcare cannot provide everyone with the same requirements. Cutting-edge technologies and excellent infrastructure are not affordable or approachable to all^2^. Nowadays, the most common and significant sector is Healthcare. An Internet of Things (IoT) enabled healthcare device predicts several diseases such as chronic kidney malady, breast cancer, skin disease, heart disease, and so on. It is feasible to stop a disease by remotely viewing it, making it possible for speedy and affordable medical treatment in contemporary healthcare systems. Machine learning and IoT-based solutions become more effective because of the advancements in machine intelligence, spectrum utilization processing, and sensing^3^. The cheap and tiny medical sensing devices are provided based on the development of microelectronics. For various diseases, the best medication, early detection of diseases, and prevention are significant attention^4^. The need for healthcare systems that can monitor and control patients’ vital signs in real time while also being inexpensive, scalable, and energy efficient is on the rise.

Technological enhancements have been helpful in everyday life, and users are also interested in using edge devices to monitor their health^5^. The smart edge or IoT devices can be easily worn in one’s hand or embedded in one’s clothing. Smartwatches, wristbands, and rings are some of the sensors worn as accessories. Heart rate measurement devices can predict heart disease during exercise and rest. Different wearable sensors normally measure the Heart Rate via electrocardiography (ECG) or photoplethysmography (PPG), which computes the heartbeat at specified intervals utilizing a machine-learning algorithm^6^. The heart rhythm can be continuously monitored via chest wrap monitors and ECG patches. However, most users are mainly interested in wearing smartwatches. The smartwatches can serve as a single lead ECG, and the back size and side of the watch serve as positive and negative electrodes. Atrial fibrillation can be noticed via these tools^7^. Radiation levels and costs increase due to traditional wireless communication technology in healthcare systems. Nevertheless, the real-time trusted health monitoring paradigm never involves radiation and provides flexible communication pathways^8^. Using the example, data transmission and communication are expected to link the local route. Data transfer and wireless network connectivity are made possible by portable medical equipment. Depending on whether individuals are inside or outside, The reliable decisions are handled by utilizing machine learning or based on deep learning algorithms.^9^. Deep learning and machine learning are the most often used paradigms in IoT-enabled healthcare systems^10,11^.

Researchers have used a number of variations of deep learning based techniques over the years, including fact and aspects of convolutional neural networks (CNN)^12^, long and short-term memory (LSTM)^5^, and connectively recurrent neural networks, along with machine learning aspects like support vector machines (SVM), random forests (RF), Naive Bayes algorithm^13^, and decision trees^14^. However, these conventional methods have caused a myriad of issues, including a lack of sufficient medical data, data threats with higher costs, improper and inadequate security, vanishing gradients, higher computational complexity and costs, and a lack of support for gradual analysis, and others^15–18^

One unique aspect of the proposed IoT-SHMF using Deep Convolutional Neural Network-based Archimedes Optimization (DCNN-AO) is its integrated approach to predicting the occurrence of heart disease in real-time while maintaining a secure IoT environment. In contrast to traditional healthcare monitoring systems, this framework combines the power of DCNN for feature extraction with the adaptive hyperparameter tuning of the Archimedes Optimization Algorithm. Traditional systems either rely on static machine learning models or do not have strong security. This integration boosts computational efficiency and generalizability, thereby increasing diagnostic accuracy. Furthermore, the technology addresses important worries about healthcare data privacy and cyber dangers by ensuring secure data transfer between IoT devices and cloud infrastructure. The framework is ideal for use in smart healthcare systems because it focuses on improving predictive modeling and ensuring secure real-time monitoring, which is a significant improvement over previous methods.

To address these issues, we put out a brand-new, safe IoT-based healthcare monitoring approach and the major contribution of this paper is summarized as follows:

- To perform authentication via registration, login, and verification of patient’s data
- The matrix-based RSA encryption method handles secure data transmission thereby enabling both data encryption and decryption.
- The blockchain network, which protects the sensor module and reward distribution, dramatically enhances user data privacy.
- The hyper parameters of a DCNN were first tuned using patient records and Archimedes Optimization Algorithm (AOA). The proposed model also guards against the over fitting of real-time input data with superior performance measures.
- DCNN-AO algorithm effectively predicts heart disease and provides an alert message to the patient’s mobile phone.

The articles continuous by covering : Section 2 delineates the literature survey of the IoT-based healthcare monitoring model, Section 3 delineates the proposed methods and the experimental results in Section 4. Section 5 Concludes the article.

## Related prior work

A smart healthcare IoT system with probabilistic image encryption and a secure surveillance technique was developed by Khan et al.^19^. The important image frames are extracted using a well-organized key frame extraction approach. Effective probabilistic techniques and lightweight encryption were developed. The testing results showed that SMSH (Secure surveillance Mechanism on Smart Healthcare) was more reliable, secure, and executed faster than other modern systems, however, there was a lack of sufficient medical data. Manogaran et al.^20^ introduced the grouping and choosing architecture with meta fog redirection (GC-MFR) architecture for secured smart healthcare alerting and monitoring. Using big data methods like Apache HBase and Apache Pig, meta fog redirection collects and stores sensor data from several sensor devices. Heart disease is successfully predicted using map-reduce. Several indicator metrics are accuracy, sensitivity, F-measure and throughput, were used to demonstrate the usefulness of the GC-MFR design. Along with increasing detection accuracy, higher data dangers and costs were identified.

The datasets related to public healthcare were utilized and saved in the cloud, and the system was created utilizing a several machine learning techniques. The system could recommend drives because of empirical and historical data stored in the cloud. Public databases were searched for diseases, including thyroid, heart, diabetes, thyroid dysfunction, dermatology, and breast cancer. The accuracy of this model was 97.26%; however, it lacked sufficient security at the lowest cost. Wu et al.^21^ proposed the deep learning (DL) model for IoT-enabled real-time health monitoring systems. Different deep-learning techniques were used to extract the important data, and wearable medical devices were used to assess the vital signs. Several statistically-based performance measures, including F score, AUC, recall, precision, and accuracy, validate the efficacy of the DL model. Although this approach reduced overfitting and increased accuracy, it had some serious drawbacks, including higher computational complexity, expense, and disappearing gradient.

Safa et al.^22^ suggested the Health Care Big Data Analytics Model (HCBDA) for optimized QoS in heart attack prediction engaged with IoT devices. The HCBDA model tracks patients’ vitals and anatomy in real time to foretell potential health problems. To conduct healthcare-related analyses, the model has located the data source by identifying potential pathways to get there. Various pathways are available to transmit the measured blood sugar values, temperature, and blood pressure. The patient-attached sensor starts the transmission with the monitored findings, and the reliable network composed by using following nodes: sensors and reliable IoT devices. The monitoring system receives the monitored results via a network of intermediary nodes; from there, it draws on big data to provide intelligence. The Trusted Forwarding Weight (TFW) and the Trusted Carrier Weight (TCW) play a role in the route selection process. Each time a packet is received, its characteristics are extracted, and the values collected are sent to the system that makes the final decision.

Shahid Mohammad Ganie et al.^23^ proposed an improved boosting-based ensemble method for identifying heart diseases. Discovering the features of data samples concerning descriptive and inferential statistics is the goal of exploratory data analysis. Pursuing these aims, the study used an interquartile range analysis to spot and replace outliers, and then used imputation to fill in missing data. Both the pre-and post-data preparation procedures were documented. Gradient boosting outperformed all other methods with a 92.20% accuracy rate for the suggested model. Gradient boosting improved the specific model’s accuracy, recall, and f1-score. It uses transfer learning to achieve higher prediction performance than previous efforts and may be applied to other illnesses with similar characteristics.

Afroj Alam and Mohd Muqeem^24^ recommended the Chaos Game Optimization based Recurrent Neural Network (CGO-RNN) for optimal heart disease prediction. The suggested method employs the Kernel Principal Component Analysis (KPCA) strategy for decreasing computational complexity and data dimensionality, and features are retrieved to classify cardiac samples for early and accurate prediction. The experimental findings showed an increase in performance of 98.99%, 98.97%, 98.95%, 98.56%, and 98.54%. This proves that the suggested approach is more effective and can accurately forecast the occurrence of heart disease.

S. Ramchandra Reddy and G. Vishnu Murthy^25^ discussed the Particle Swarm Optimization and Neural Network for Cardiovascular Disease Prediction. The PSO-NN framework improves prediction accuracy by handling data imbalances using a customised cost function and missing data with mean replacement approaches. It also uses feature importance techniques to choose the most effective features. Based on these findings, PSO-NN may be trusted to detect CVD early in practical settings.

**Table 1:** Literature survey of IoT-based healthcare monitoring model.

Author	Methods	Datasets	Platform	Advantages	Limitations
Khan et al.^19^	SMSH	Wider-face dataset	MATLAB simulation	Better security with minimum execution time	Lack of adequate medical information
Manogaran et al.^20^	GC-MFR	Cleveland heart disease database	JAVA	Better detection accuracy and minimum task completion time	Data threats with higher cost
Kaur et al.^26^	Random forest (RF)	Public datasets	WEKA open-source tool	97.26% accuracy and minimum cost	Improper security
Wu et al.^21^	Deep learning	Real-time dataset	MATLAB	Minimum overfitting and enhanced accuracy	Vanishing gradient, higher computational complexity, and cost
Zhu et al.^27^	Edge-fog computing framework	Gliomas datasets	Azure cloud	Smaller latencies with higher accuracy	Does not promote gradual analysis
Juyal et al.^28^	AI-enabled cloud-based IoT	Skin image dataset	MATLAB	Improved accuracy and detection rate	Lots of security threats

Zhu et al.^27^ suggested an Edge-fog computing framework for IoT-enabled glioma disease management. The model is set up to work with certain operational models, such as those based on user demands, service quality, expected accuracy, and precision. Performance metrics including execution time, accuracy, latency, and power consumption, are used to assess the recommended approaches’ effectiveness. Although the edge-fog computing architecture has better accuracy and shorter latencies, it does not support developed prediction models or incremental analysis. AI-enabled cloud-based IoT was introduced by Juyal et al.^28^ for smart skin health monitoring. Both preventive and diagnostic model framework was offered. This model is implemented using MATLAB software. Even though there were several security concerns, this gave better accuracy and detection rate. The literature survey of IoT based healthcare monitoring model is tabulated in Table 1.

## Methods

### Preliminaries—archimedes optimization algorithm

Mathematical principles from physics form the basis of the Archimedes Optimization Algorithm (AOA). It is with the densities and accelerations that the AOA initiates the initial object population and random volumes. This is the mathematical model of the AOA stages:

### Initialization

Equation (1) describes the initialization of every object position.

1 $$\begin{aligned} OBT_j=Lower_j + r{\times } (Upper_j-Lower_j); j=1,2,...,L \end{aligned}$$

Where ${Upper_j}$ and ${Lower_j}$ are the upper and lower boundaries, and is the j$^{\text {th}}$ population of M objects. Equations (13) and (14) are used to update the volume (Vol) and density (Den)^29^.

2 $$\begin{aligned} & Den_j=R \end{aligned}$$

3 $$\begin{aligned} & V_j=R \end{aligned}$$

The D-dimensional vector is created by using a random (R) interval of 0 to 1. The following equation is used to update the j$^{\text {th}}$ items with the acceleration (ACL).

4 $$\begin{aligned} ACL_j=Lower_j + R{\times }(Upper_j-Lower_j) \end{aligned}$$

By analyzing the starting population, choose the objects with the best fitness values. The values $\text {Best}_{\text {ACL}}, \text {Best}_{\text {Vol}}, \text {Best}_Y$ have been assigned.

### Volume and density updating

During the T+1 iteration, the volume and density of the jth item are updated using Eqs. (5) and (6)^30^.

5 $$\begin{aligned} Den_j^{T+1}&= Den_j^T + R \times (Den_{\text {best}} - Den_j^T), \end{aligned}$$

6 $$\begin{aligned} Vol_j^{T+1}&= Vol_j^T + R \times (Vol_{\text {best}} - Vol_j^T). \end{aligned}$$

The optimal volume and density are $\text {Den}_j^{T+1}$ and $\text {Vol}_j^{T+1}$.

### Density factor and transfer operator

After a short period of time, the objects attempt to achieve balance and collide. The transfer operator $\text {TO}$ is used to implement the converts search space from exploration to exploitation.

7 $$\begin{aligned} |TO| = \exp \left( \frac{T - \text {Max}_T}{\text {Max}_T}\right) \end{aligned}$$

Anywhere, $\text {T}$ and respectively, denote the average and a maximum number of iterations. The element that reduces density is $\text {Den}_M.$

8 $$\begin{aligned} Den_{M}^{T+1} = \exp \left( \frac{T-Max_{T}}{Max_{T}}\right) - \left( \frac{T}{Max_{T}}\right) \end{aligned}$$

### Exploration stage

Things can collide as a result of $T0 \le 0.5$. For each repetition of $T+1$, Equation (9) modifies the object acceleration.

9 $$\begin{aligned} ACL_j^{T+1} = \frac{Den_{j,\text {min}} + Vol_{j,\text {min}} \times ACL_{j,\text {min}}}{Den_j^{T+1} \times Vol_j^{T+1}} \end{aligned}$$

These three variables–velocity, density, and random material properties–are expressed as $\text {Vol}_{Rm}$,$\text {Den}_{Rm}$ and $\text {ACL}_{Rm}$. During the third iteration, the exploration is guaranteed.

### Exploitation stage and normalize acceleration

The number $T0 > 0.5$ ensures that the objects do not collide. The best object acceleration is shown by the symbol $\text {A}_{best}$.

10 $$\begin{aligned} ACL_{j}^{T+1} = \frac{{Den}_{best} + {Vol}_{best} \times {ACL}_{best}}{Den_{j}^{T+1} \times Vol_{j}^{T+1}} \end{aligned}$$

The normalizing acceleration percentage is determined using the following formula.

11 $$\begin{aligned} ACL_{j-Normalizing}^{T+1} = \chi \times \frac{ACL_j^{T+1} - ACL_{min}}{ACL_{max} - ACL_{min}} + \lambda \end{aligned}$$

The normalization range is $\chi$ and $\lambda$ in this equation.

### Updating position and validation

The mean object position $T0 \le 0.5$ is updated using the formulae below, which are based on the exploration stage.

12 $$\begin{aligned} Z_j^{T+1} = Z_j^T + K_2 \times R \times ACL_{j-normalizing}^{T+1} \times Den \times (Z_R - Z_j^T) \end{aligned}$$

K is a constant word. Update the object position $T0>0.5$ using the equation below based on the exploitation stage.

13 $$\begin{aligned} Z_j^{T+1} = Z_{best}^T + flag \times K_2 \times R \times ACL_{j-normalizing}^{T+1} \times Den \times (T \times Z_R - Z_j^T) \end{aligned}$$

The direction of motion of the flag is changed.

14 $$\begin{aligned} flag = {\left\{ \begin{array}{ll} 1 & \text {if } I \le 0.5 \\ -1 & \text {if } I > 0.5 \end{array}\right. } \end{aligned}$$

As a result, $I=2 \times R-K_4$. Each item is measured through a defined objective function, $Best_{ACL}$, $Best_{Vol}$, $Best_{Den}$ and $Best_Z$ are assigned.

Cloud computing has several advantages, such as better scalability, centralized model updates, and the ability to use high-performance GPUs and TPUs for deep learning inference, greatly increasing prediction throughput and decreasing processing time. Interaction with distributed storage systems can safely handle long-term health records and massive patient information. For time-sensitive applications like emergency warnings, the main benefit of an edge computing environment is the real-time responsiveness and decreased latency that results from processing data closer to the data source. Along with the blockchain and Matrix-based RSA encryption, it improves data privacy by reducing the amount of sensitive health data sent to third-party servers. However, there are a few drawbacks to consider. One is that heavy DCNN operations might lead to higher energy consumption and thermal limits on edge devices. To keep things efficient, it may be necessary to implement model quantization or pruning. Problems with real-time performance might arise in cloud environments due to data transmission delays and reliance on reliable internet access. Cloud communication security measures should also include strong encryption and access control rules to prevent threats like man-in-the-middle attacks. Moving to the cloud or an edge environment improves practicality but requires optimization to balance latency, security, and accuracy.

### Proposed work

**Fig. 1:**
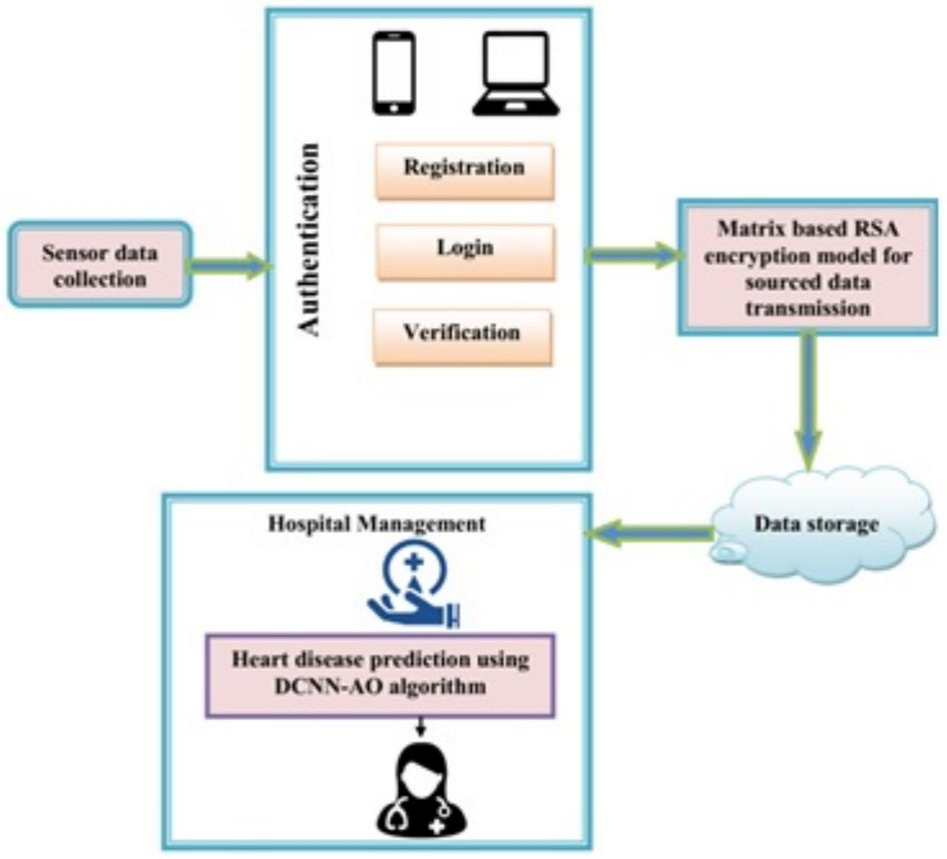
Overall architecture of the proposed model.

Early detection of heart disease can greatly improve the prognosis and chances of survival, allowing patients to receive proper medical treatment. Accurate heart disease diagnosis, as well as security maintenance, is a major concern for an effective system predicting heart disease. Figure 1 describes the outline of the proposed model architecture.

### Authentication

Authentication is the first step. Assets of the smart terminal focus on a small number of mobile applications. Information gathering and uploading are among its main responsibilities. The three main steps in authentication are registration or listing, logging in, and verification^31^. Each of them is delineated as below:

### Registration

The hospital or healthcare applications consist of patients’ information based on registration. The administrator must permit a system before a user may utilize it. After registration, the supervisor provides the information for authentication.

### Login

If a user attempts to log into the system, the user must grant the authentication details provided by the administrator^32^. The user will send the login information, which includes the username and a partial key that serves as the password. The user inputs and submits their username as well as their password.

### Verification

Users can access the system, which is intended for any vital information. The blockchain administrator collects and merges all dispersed keys, which the blockchain administrator only creates a key for safe key transfer^33^. If the user’s information matches, the corresponding user is added in the cloud storage. Otherwise, the process goes back to registration stage.

## Secure data transmission and authorization

In this section, the Matrix-based Rivest–Shamir–Adleman (RSA) encryption method handles secure transmission of data and authorization, as depicted in the next sub-section. Various technologies like economic models, consensus algorithms, cryptography, and mathematics are present in the blockchain. Each record in the block model of a transactional database is saved using a secure distributed ledger. Both peer-to-peer networks and consensus mechanisms offer distributed data synchronization^34^. The block is mostly made up of the header and data. As a result, the header contains the nonce, Merkle root, timestamp, current block, and preceding block information. Based on the Merkle tree, the main component of blockchain technology, the data content with its security authentication is accelerating. Pairing two transactions together and hashing them produces the hash. Each IoT node is validated in the IoT network using the Merkle tree without downloading and verifying the complete block. The Merkle root is stored in the block header, allowing each network node to confirm the transaction.

We used the Matrix-based RSA encryption method to handle secure transmission of data and authorization. While the idea of storing verified IoT data on the blockchain is appealing, its centralized nature often leaves it susceptible to external threats. As a result, the encryption approach is employed. This project handles storage using a decentralized interplanetary file system (IPFS). To construct the Merkle tree, data is encrypted and then partitioned into several blocks before being saved in the IPFS file system. The core idea behind this proposed approach to enhance IoT privacy is integrity verification, which makes use of matrix-based RSA encryption technology^35^. This technique has the potential to detect tampering with the Internet of Things data stored in the blockchain database by either blockchain members or cloud servers. Each transaction reflecting data produced by IoT-based health monitoring equipment is cryptographically linked to previous entries and stored immutably using a decentralized ledger structure. This prevents unauthorized tampering or manipulation of the data. Access control restrictions may be enforced using smart contracts, ensuring that only authorized healthcare practitioners or stakeholders can access or make changes to sensitive medical records. In addition, data is protected before it reaches the blockchain via Matrix-based RSA encryption, which provides an extra strong level of anonymity throughout transmission and storage.

### Key generation

where $o=x *y$ in which x and y create two large prime numbers.From $MJ(a, \psi (k))$ select invertible matrix I. Take $(r, \psi (k))$ and (K, A, I) as the private and public key from $r = I^{-1} \left[ \mod \psi (A) \right]$. For key generation, randomly selects the encryption matrix. The matrix A determines the number of data blocks. Even without the knowledge of$\psi (k)$ determine the factor. For solving $I^c = I_2 (\mod K)$ the smaller integer values are selected. Identify matrix $I_2$ in which s-fold application of exponentiation $(\Lambda \Lambda )$ encrypts IoT data and is decrypted using $I^{c-1}$

15 $$\begin{aligned} \text{ D }A \Lambda \Lambda I^{c}= \text{ D }A(\Lambda \Lambda I_2) = DA(\mod K) \end{aligned}$$

To prevent the disclosure of sensitive information, verify each value of ${I^c}$. It is the responsibility of the encryptor to ascertain if the randomly chosen matrix is invertible.

### Encryption

The invertible matrix I encrypted with (A-1) different blocks (X1, X2,...., XA-1) for each new digital data block (Ym), which is given as follows:

16 $$\begin{aligned} \left( I_{1}, I_{2}, \ldots , I_{n-1}, I_{n}\right) ^{r} = \left[ (X_{1}, X_{2}, \ldots , X_{n-1}, Y_{n})^T \Lambda \Lambda \right] (\mod K) \end{aligned}$$

Combine data from previous cycles with past encryptions. Encrypt and confound the encrypted data.

### Decryption

From the sender, receive n encrypted data blocks r every set. The matrix F calculates the decryption process.

17 $$\begin{aligned} \left( X_{1}, X_{2}, \ldots , X_{A-1}, Y_{n}\right) ^{T} = \left[ (I_{1}, I_{2}, \ldots , X_{a-1}, I_{a}) \Lambda \Lambda \right] ^T (\mod K) \end{aligned}$$

Integrating the key generation center, cloud server, data owner, and third-party auditor (TPA) into blockchain-based data storage allows the Internet of Things (IoT) node to preserve its data. The IoT user sends their data to a blockchain-enabled cloud server. Following data storage, the user is required to sign a verification contract with the TPA. When it comes to data, the TPA is the one to trust^36^. An affiliation between the cloud server and the cloud service provider (CSP) allows the CSP to offer storage services. It provides server power for processing and data storage. Giving back to the user is the main focus of the TPA, and it accomplishes this by communicating verification findings to the user and the server in the cloud. There is an instantaneous detection of data corruption. All interactions between the TPA and any entity are verified. An incomplete private key is generated by the Key Generation Center (KGC) under the jurisdiction of the authority. This center uses the user’s identification. The blockchain utilizes all four matrix-based RSA encryption parameters, which are listed below:

- *Data storage* This method enables the data storage to be outsourced to the cloud. In order to assist the TPA for ensuring the data integrity, the IoT node needs to sign the data. The server sends back a confirmation message after cloud storage of the data is successful.
- *Audit* The data integrity check and assisting the cloud server in efficiently determining outsourced data is audited by the TPA using the Matrix-based RSA encryption technique.
- *Log generator* This step assists the TPA in creating the log file that contains the TPA’s verification information.
- *CheckLog* In order to analyze the TPA’s behavior, the Matrix-based RSA encryption approach computes the accuracy and validity of the user’s log file.

**Fig. 2:**
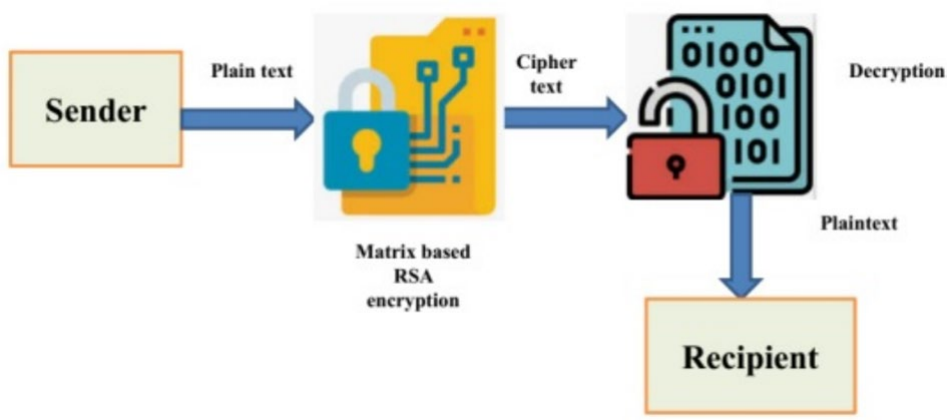
Flowchart of the matrix-based encryption key for secure communication.

The dangers arise in the adversarial paradigm, which comprises dishonest auditors, malicious users, and partially trustworthy servers. Data theft can be concealed by a semi-trusted organization, such as a cloud server, by providing false evidence information to fool the TPA. However, while the cloud server may be able to modify the public key of an Internet of Things device, it will not have access to the master key of the KGC. Despite being able to replace its master key in some situations, the KGC is unable to access its public key. On rare occasions, the TPA might act inappropriately by delaying the completion of the verification. The compromised TPA could potentially crash into the cloud server, resulting in undetected data corruption. In certain cases, hostile Internet of Things (IoT) nodes can upload encrypted health data to a server in the cloud, which puts patients at risk. Figure 2 expresses the flowchart description of secure data communication using matrix-based encryption. A structural improvement over classic RSA, matrix-based RSA uses matrix operations to encode data blocks, allowing for increased encryption speed via parallelization of calculations. This technology enhances scalability in IoT applications without substantially increasing computing complexity or key length by enabling concurrently processing multi-dimensional data, such as time series, from wearable sensors. When hardware acceleration is available, such as GPU or SIMD support, matrix-based RSA may go faster than classical RSA since matrix operations are optimized for these architectures. Conversely, matrix-based RSA usually has greater computing overhead for key generation and encryption at the same security level as more modern algorithms like Elliptic Curve Cryptography (ECC) or lattice-based schemes, especially for devices with limited power at the edge. For instance, ECC is more efficient for large-scale IoT installations because it provides equal security with lower key sizes and quicker execution on restricted devices.

### Cloud server based on the hospital

The data is safely retrieved and consolidated on the public cloud server. The health cloud’s structural design includes many patients’ health data. The detected values of the various patients were then downloaded to the Hospital’s specialized cloud server^37^. As a result, the downloaded information is decrypted. The decryption technique makes use of the distributed key. The decryption process is the inverse of encryption, and the decrypted data is expressed as follows,

18 $$\begin{aligned} Decrypteddata_S=\left\{ Dd_1,Dd_2,Dd_3,\ldots ,Dd_m\right\} \end{aligned}$$

The m-number of decrypted values is signified as $Dd_m$ and the decrypted dataset is $Decrypteddata_S$.

### DCCN-based AO algorithm for heart disease prediction

The proposed DCNN-based AO method to predict heart disease is discussed in this section. The section below describes the heart disease prediction model.

### Deep convolutional neural network

The several layers that make up a convolutional neural network (CNN) include the following: input, softmax, batch normalization, convolution, class output, and completely connected. The neural network’s first and input layer is responsible for receiving the raw data. Consistent with the input layer size, the data is input^38^. The feature diversity is supplied by a variety of convolution kernels of slides, filters, or convolutional layer kernels, and different local information is recorded in this way. The values are chosen by the learners during the training process. It is the feature map that determines the padding, stride, filter size, and filter count.

### Heart disease prediction using DCNN-based AO algorithm

**Fig. 3:**
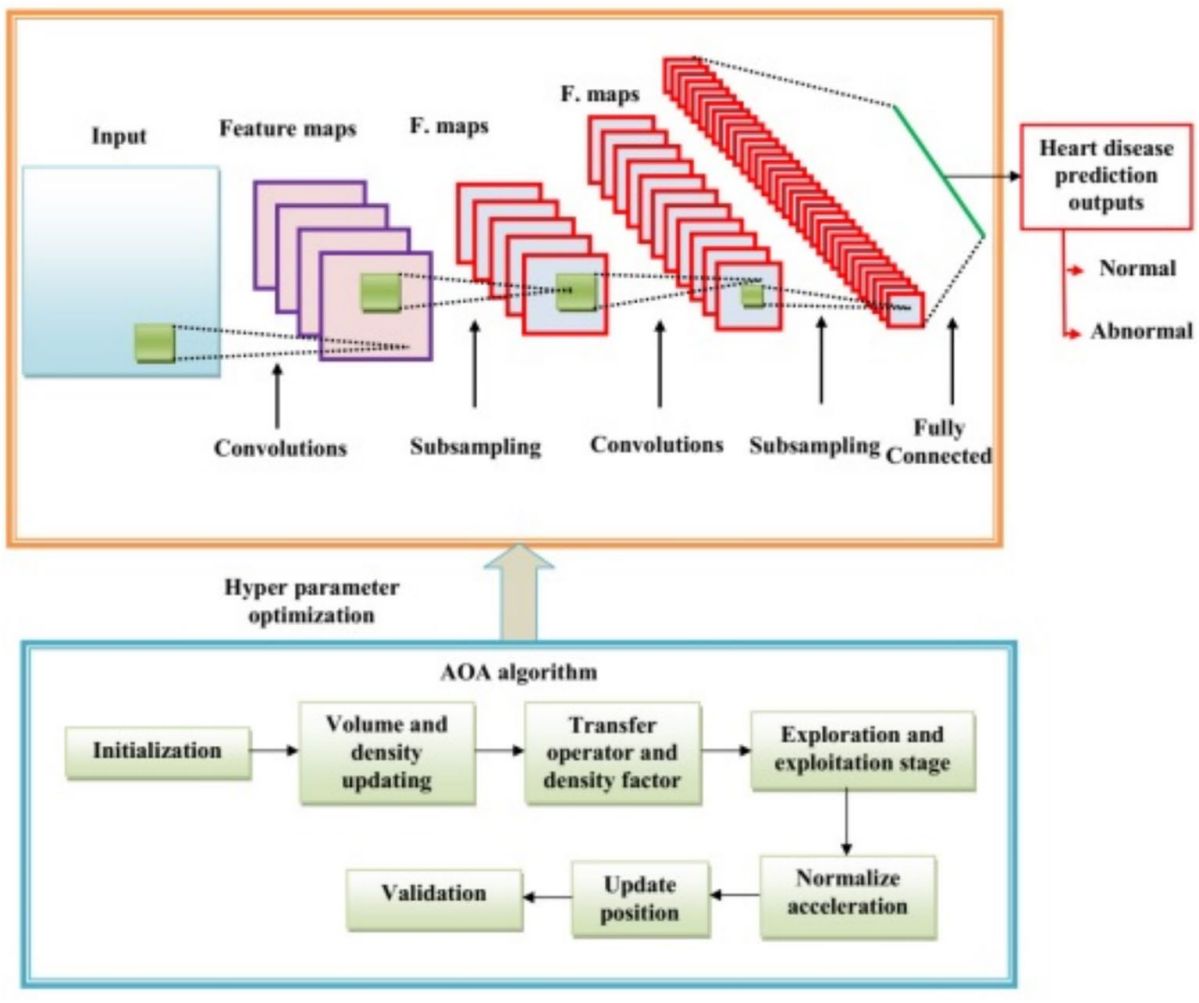
Heart disease prediction using DCNN-based AO algorithm.

A total of 100 neurons in present in the dense layer at the end of the architecture. The heart disease prediction using the DCNN-based AO algorithm is illustrated in Fig. 3. In many practical situations, the distribution of data in a dataset is not uniform. Most cases of cardiac disease detection have imbalanced data^39,40^. The objective is to encourage more representation of underrepresented groups in designations.

The efficacy of accuracy (and error rate) in assessing a classifier’s performance is an evident difficulty that stems from the class imbalance problem. This is because most conventional classifiers focus on accuracy optimization and produce models similar to the naive model mentioned before. Although accurate, such a classifier is worthless in most real applications since the minority class is frequently the class of interest (otherwise, a classifier would not be required, as the class of interest occurs practically always). As a result, several strategies have been created to address the class imbalance issue. These techniques may be divided into two broad categories: sampling and skew-insensitive classifiers. The current machine learning approach becomes increasingly skewed toward the majority classes as the number of classes increases. The misclassification of minority classes occurs frequently. The DCNN-based AO model is utilized to solve this problem, and it is not biased toward the majority classes as the number of samples grows. The DCNN’s accuracy improves as the number of cases rises, not declines. Finally, the DCNN-based AO algorithm distinguishes between groups with typical and atypical heart disease. Based on this defined result, the hospital’s administration determines whether or not there is heart disease. If the data analyzed contains significant abnormalities, the HM sends an alarm message to the patient’s mobile phone.

Following pre-processing, feed the pre-processed data into the DCNN-AO algorithm for heart disease prediction. DCNN is an excellent classification approach that enhances accuracy. However, the increased number of layers, neurons, dropout rate, and other hyperparameters hampered the CNN’s performance^41^. For DCNN hyperparameter optimization, we used the Archimedes Optimization (AO) algorithm in this study. The AO algorithm outperforms others in terms of convergence speed, searchability, the ability to exploit and explore, efficiency, and computational time, among other factors. The AO algorithm effectively optimizes the CNN hyperparameters in this study, resulting in improved classification results. Maximum accuracy is considered the fitness value. We looked at challenges like CNN hyperparameter tuning (HT) with improved prediction performance (CP), both multi-objective optimization problems.

19 $$\begin{aligned} & CP(HT)=(CP_1(HT),CP_2(HT), \dots ,CP_k(HT)) \end{aligned}$$

20 $$\begin{aligned} & Subject to: U(HT)=(U_1(HT),U_2(HT),\dots ,U_j(HT)) \end{aligned}$$

21 $$\begin{aligned} & HT_i^{F}\le HT_j \le HT_j^{V} \end{aligned}$$

**Fig. 4:**
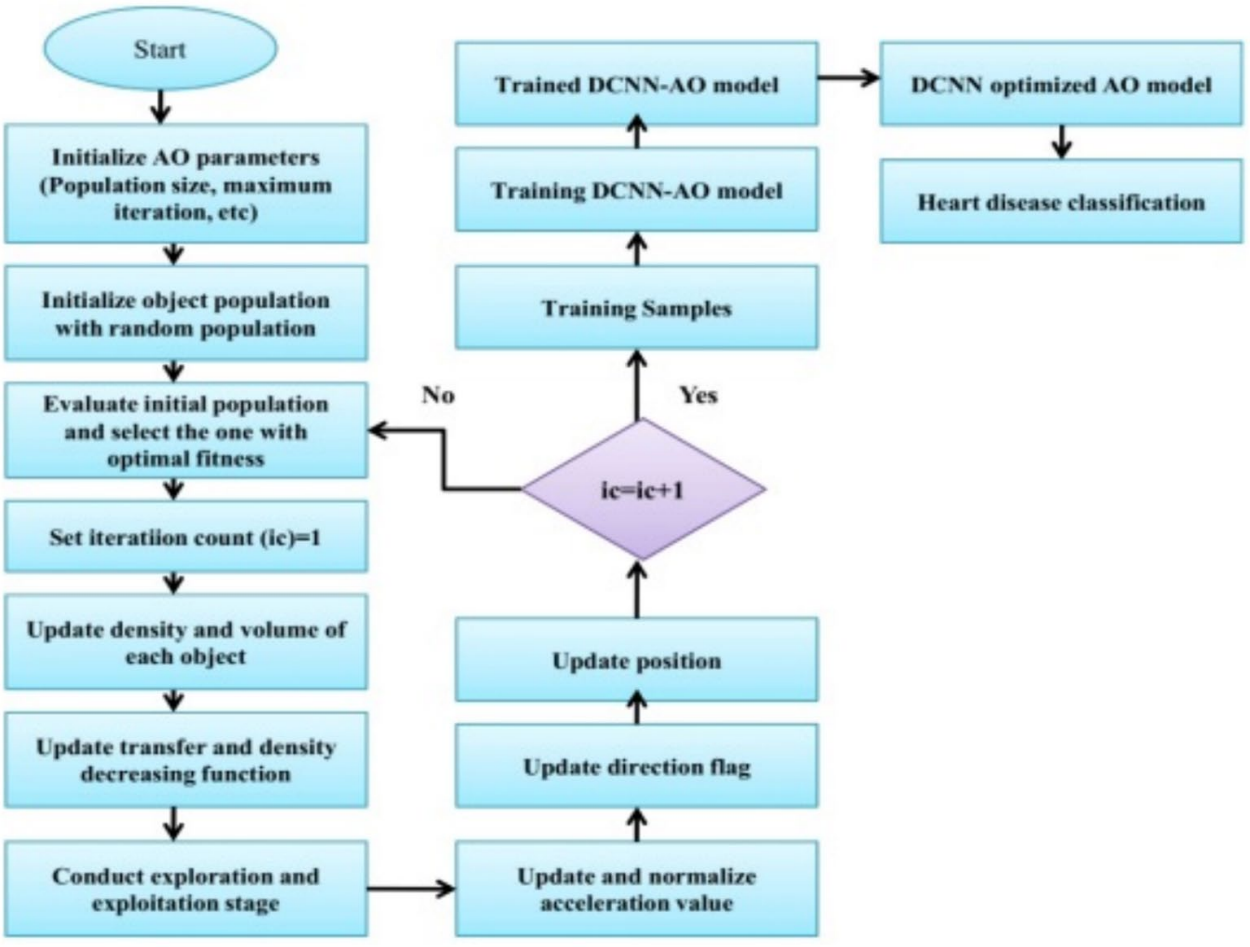
Flowchart of the AO algorithm for optimizing the DCNN architecture.

Inside the parameter space, the design variable vector has $HT_i^{F}$as its upper boundary and $HT_j^{V}$ as its lower boundary. Where U represents the fitness of the parameters, the vector representation of the objective space and the target function must be maximized^42^. The hyperparameters are vital in improving the CNN’s performance and mainly depend on parameters such as regularization coefficient, number of epochs, momentum, etc. Table 2 displays the parameters that were optimized by the AO algorithm. An illustration of the AO algorithm’s flowchart for DCNN architecture optimization can be shown in Fig. 4. Neural networks use a regularization technique called dropout to prevent overfitting. A random subset of neurons is “dropped out” during training by having their outputs set to 0 with a certain frequency. Because it can no longer rely on certain neurons, the procedure encourages the network to acquire more reliable and independent features. Dropout enhances generalization while lowering the likelihood of overfitting. The Dropout in the model is given a value of 0.2 to avoid overfitting. The DCNN architecture is good at automatically finding important features from raw sensor inputs, such as ECG signals or vital signs. This allows for capturing spatial and temporal relationships crucial for accurate heart disease prediction. Hyperparameter optimization, which includes learning rates, convolutional filter sizes, and dropout ratios, is crucial to DCNN success. To solve this issue, we used the Archimedes Optimization method, which works like how things float in water to effectively search through hyperparameter options, helping us avoid getting stuck in less optimal solutions and speeding up the process.

**Table 2:** DCNN hyperparameters optimized using the AO algorithm.

Hyperparameter	Description	Optimized value
Learning rate	Controls the speed of the gradient descent algorithm	0.0001
Momentum	Controls the impact of the previous weight update	0.900
Number of epochs	Determines how many times the training dataset parameters will be updated	30
Regularization	Overcomes the overfitting issue	0.2

## Real-time simulation and computational complexity analysis

This is especially important for older people, who require particular medical attention and round-the-clock supervision. The amount of heart disease-related fatalities that go unreported is a further cause for worry. This research can develop a system that foretells cardiac disease. To achieve this, we must look at the risk factors for heart disease, such as blood sugar levels, age, cholesterol, and chest discomfort. Physiological sensors and a microcontroller can create a system that can avert health-related catastrophes for a continuous health monitoring system. Consequently, we suggest a smartphone-based approach for monitoring and forecasting heart disease. The DCNN-AO method applies not only to the elderly but also to neonates, adults, and stroke patients in terms of anticipating cardiac illness. Three challenges must be acknowledged. Data pre-processing is required initially. Everything in the input signal that does not contribute information to the model must be deleted. Furthermore, ensuring that the data gathering is spread consistently will be critical. To do this, we generated new data that enabled us to train the model using a set of balanced class data. Without communication-induced delays in real-time prediction, it was assumed that all IoT sensor nodes functioned under stable network circumstances with dependable data transfer rates and low latency. Secondly, it was assumed that the patient data used to train and test the DCNN-AO model was clean, uniform in sampling frequency across all input parameters, and reflective of real-world clinical situations. Lastly, it was presumed that the Matrix-based RSA encryption and blockchain modules did not impose any substantial processing burden that may impact the system’s real-time performance.

The k-dimensional patient records the proposed model processes from the database serve as the primary gauge of the computational complexity of the DCNN-AO method. The cost of protecting the patient data is O(l2) using matrix-based RSA encryption operations. O(k*l2) is the storage cost for sending k patient records to the cloud. The DCNN-AO method’s execution duration is mainly affected by three variables: the maximum number of iterations (M), the dimensionality of the optimization problem (t), and the number of steps in the problem (r). Consequently, the DCNN-AO method’s total temporal complexity looks like this: In terms of space complexity, the DCNN-AO architectural model is O(z), where z is the space related to starting the dataset’s instance count, according to the heart disease index.

## Results and discussion

This section discusses our proposed approach and presents its results within a broader framework. The transactions were mainly conducted using a local blockchain run by Ganache, which offers 10 free accounts and the encryption and decryption keys associated with the accounts. The connection to the adjacent Ethereum network is offered via the metamask software, and the patient’s records are uploaded with the help of a connected IPFS node. The smart contract is developed using the solidity programming language. The IoT sensors used to assess the risk of heart disease in healthy individuals are an accelerometer, Barometer, Global Positioning System, Photoplethysmography device in the form of a smartwatch or band, ECG chest wrap device, and oscillometry sensor for measuring blood pressure with a wrist cuff. The proposed model was implemented in Python 3.5 using the Keras and pip3 libraries. Table 3 explains the parameter settings. By incorporating patient interaction and feedback, the IoT-SHMF system can enhance its personalization and prediction accuracy through various technological methods. In addition to data collected by physiological sensors, the model may learn subjective health indicators by integrating patient-reported outcomes (PROs) via mobile or wearable interfaces. These PROs can include symptoms, medication adherence, lifestyle behaviors, and perceived health status. Using these data points as auxiliary features, and the DCNN may improve its predictions by considering patterns in each person’s health-related behaviors. The second option is to include adaptive feedback loops or reinforcement learning. In this setup, the system may constantly change the thresholds for predictions or the alarm mechanisms depending on the real-time input from patients about irrelevant or false positives. In addition, the Archimedes Optimization component may be fine-tuned using patient input to dynamically reweight the value of features, making it more sensitive to patient-specific situations.

**Table 3:** Parameter settings.

Parameters	Ranges
Population size	50
Maximum number of iterations	100
Number of input layers	10
Number of hidden nodes	20
Number of output layers	2
Learning rate	0.1
Number of epochs	32
Dropout	0.243
Batch size	8

The data are gathered from the Cleveland Heart Disease database (https://archive.ics.uci.edu/ml/datasets/heart+disease). It includes 73 attributes. The gathered data is divided into normal and abnormal. The experimental setting of different techniques used to conduct comparative analysis, such as Random Forest (RF)^26^, DL^21^, SMSH^19^, and GC-MFR^20^, is provided in Table 4.

**Table 4:** Hyperparameters and model architectures.

Parameter	Value
Random Forest (RF)^26^	
Number of trees	500
Max features	Sqrt
Deep Learning (DL)^21^	
Number of input parameters	12
Number of output parameters	1
Number of hidden layers	2
Number of hidden units in each layer	45, 35
Batch size	128
Learning Rate	0.001
Single-Shot MultiBox Detector (SSD)^19^	
Big anchor shape	[(116,90), (156,198), (373,326)]
Mid anchor shape	[(30,61), (62,45), (59,119)]
Small anchor shape	[(10,13), (16,30), (33,23)]
Architecture	Darknet
Number of layers	53
Genetic-based Multi-Feature Regression (GC-MFR)^20^	
Number of records	100
Number of generations in each map	100
Number of keys	1

These outcomes are stored in different tables, as shown below. The patient’s clinical parameters make up the crucial information and are provided in Table 5. The personal data of the patients are stored in Table 6. These are considered sensitive data. The normal patient data are stored in Table 7. The data in data centers and their details are stored in Table 8.

**Table 5:** Patient’s clinical parameters.

Patient ID	Clinical parameters	Address	Doctor details
B101	Heart rate, Body temperature, Blood pressure	Mumbai	Specialist 1
H189	Heart rate, Body temperature, Blood pressure	Delhi	Specialist 4
T456	Heart rate, Body temperature, Blood pressure	Chennai	Specialist 7

**Table 6:** Personal data of the patient.

Patient ID	Name of the patient	Age	Sex	Address	Location
U189	John	78	M	123, ABC street	Hyderabad
jO082	Gracy	67	F	67, elive street	Rajput
LI190	Neenu	23	F	956, Lan	Rajavalli

**Table 7:** Patient Information.

Patient ID	Age	Sex	Location
P016	65	M	Himachal
K1034	23	M	Rajasthan
H389-3	46	F	Manali

**Table 8:** Data storage information.

Storage provider	Geographical location	Patient ID	Data type
Microsoft	Japan	G561	Sensitive
Amazon	Canada	S2890	Normal
Google	Washington	G6123	Critical

### Performance evaluation

The proposed IoT-based heart disease prediction uses the DCNN-AO approach which can be used to predict heart disease. We have taken several parameters to analyze the performance: accuracy, precision, F1-measure, recall, security analysis, decryption, encryption, and key generation time. The proposed approach can effectively predict heart disease from the collected dataset.

#### Precision (P)

The ratio of the total number of predicted values to the number of correctly predicted heart disease cases from the dataset gathered using our suggested method is called the accuracy rate. It is possible to state it as,

22 $$\begin{aligned} P = \frac{TP}{FP+TP} \end{aligned}$$

#### Accuracy(A)

It is the degree to which actual output matches the accuracy of heart disease predictions. alternatively, it may be assessed as,

23 $$\begin{aligned} A = \frac{TP+TN}{FP+TP+FN+TN} \end{aligned}$$

True positive (TP), true negative (TN), false-negative (FN), and false positive (FP) rates for the proposed IoT-based heart disease prediction system are shown above.

#### Security analysis (SA)

This is referred to as how threat-proof our proposed framework is. That is the removal of intruders from the system or ignorance about them. It may be stated as follows:

24 $$\begin{aligned} SA = \frac{\text {Hacked data}}{\text {Original data}} \end{aligned}$$

#### F1-Measure (F)

F1-measure is defined as our proposed model’s prediction accuracy of heart disease. It can be expressed as,

25 $$\begin{aligned} F = \frac{2*(P*R)}{(P+R)} \end{aligned}$$

#### Recall (R)

It is the degree to which our suggested model successfully extracts information about cardiac diseases from the sampled dataset. This idea can also be stated as,

26 $$\begin{aligned} R = \frac{TP}{TN+TP} \end{aligned}$$

#### Encryption time

Encryption time is the amount of time it takes to convert from one pattern to another pattern of data. It is possible to state it as,

27 $$\begin{aligned} E_T = E_{end} - E_{start} \end{aligned}$$

Here, the encryption time is denoted as $E_t$. The encryption ending is denoted as $E_end$ and the starting period of the encryption is given as $E_start.$

#### Key generation time

It is the amount of time it takes for the system to produce the key needed for encryption and decryption. It can be expressed as,

28 $$\begin{aligned} K_T = K_{end} - K_{start} \end{aligned}$$

The starting time is represented as $K_{start}$ and the ending time is indicated as $K_{end}$.

#### Decryption time

It can be determined as the time required for the completion of the decryption process. It can be expressed as,

29 $$\begin{aligned} D_T = D_{end} - D_{start} \end{aligned}$$

The starting and ending times of the decryption process are indicated as $D_{start}$ and $D_{end}$ correspondingly.

**Fig. 5:**
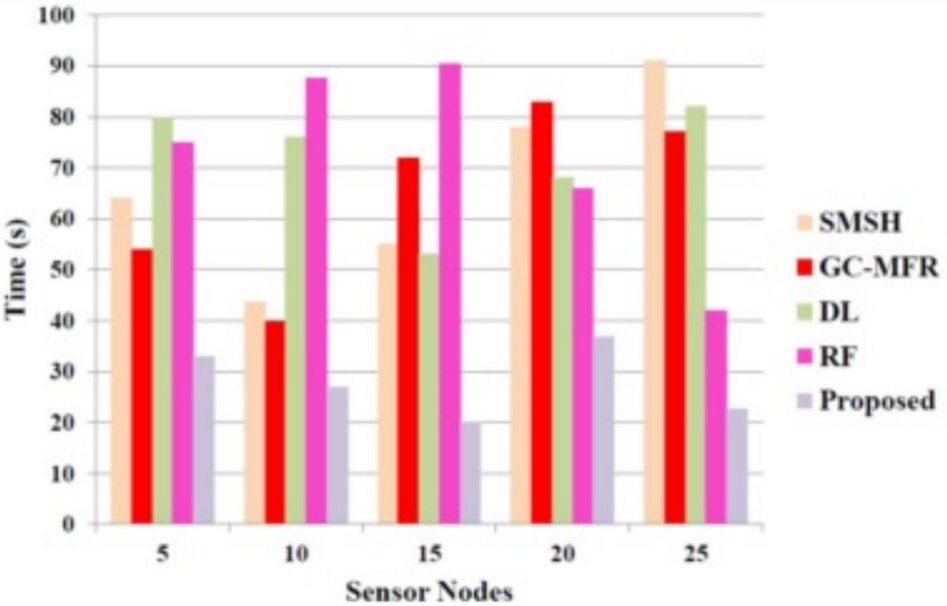
Performance evaluation based on the encryption time.

To analyze and compare the performance parameters of our proposed work, we have taken some of the previous works, such as SMSH^19^, GC-MFR^20^, DL^21^, and RF^26^. Figure 5 illustrates the encryption time while transmitting the data from one IoT node to the server. The figure shows that the proposed method achieves a low time of about 23 seconds when the sensor nodes equal 25. The other approaches take more time. Thus, our method reduces the computational complexity while predicting heart disease. Adding more diverse sensor data sources can make the prediction model more accurate and robust. These sources include continuous blood pressure monitoring, skin temperature, respiratory rate, blood oxygen saturation (SpO$_2$), and motion data. This provides a richer, more multidimensional physiological context. The DCNN-AO methodology can pick up on minor changes in patients’ health statuses and complicated interdependencies because of these other data streams that single-source inputs could miss. The model’s capacity to differentiate between symptoms similar to those of other diseases may be enhanced by multimodal feature fusion, which it can do with various inputs. This improves the accuracy of predictions and makes the system more resistant to data loss or malfunctioning sensors. Improved generalizability to different clinical situations and patient groups is a side effect of the Archimedes Optimisation algorithm’s dynamic network tuning, which allows it to extract useful characteristics from each modality.

**Fig. 6:**
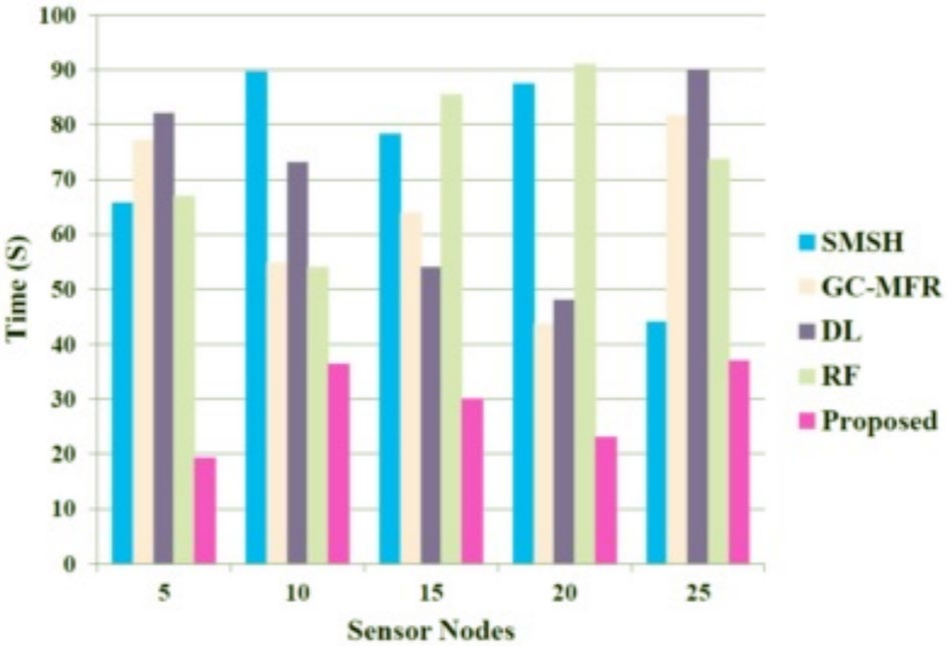
Performance evaluation based on the decryption time.

The decryption time of the proposed method and other approaches are graphically represented in Fig. 6. The figure shows that when the number of sensor nodes increases, the decryption increases; however, our proposed approach achieves low decryption time compared to others. Adopting the matrix-based RSA approach enhances the encryption and decryption speed and thus mitigates the time requirement. The decryption time of our proposed approach when the sensor nodes are equal to 25 is 37 seconds. Meanwhile, other approaches, RF, DL, GC-MFR, and SMSH, utilize higher decryption times of 74s, 90s, 81s, and 45s, respectively.

**Fig. 7:**
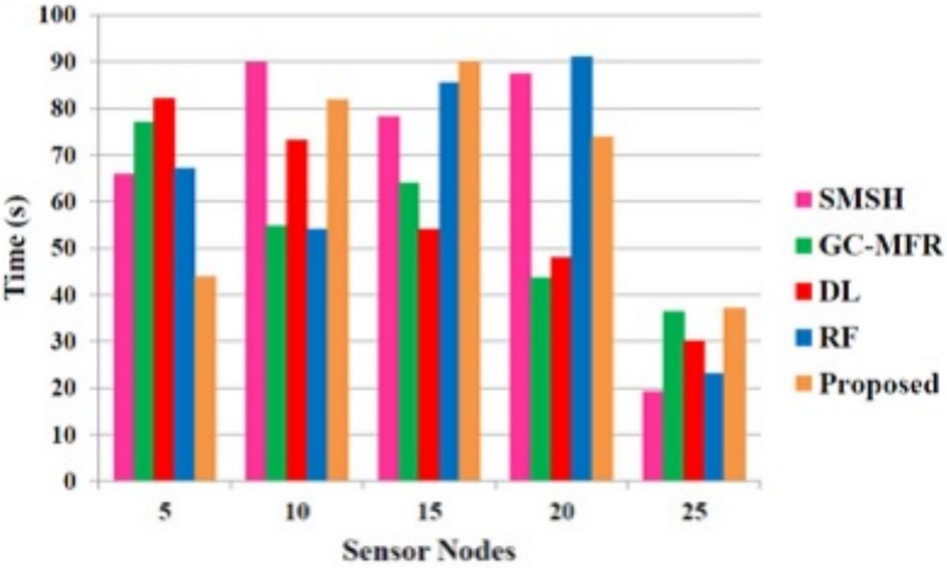
Performance evaluation based on the key generation time.

Figure 7 illustrates the performance evaluation based on the key generation time. The key is generated while transmitting the document from the hospital to the server. This will help to ensure the authentic transmission of data. Only users with an authentic key can access the stored data. Hence, it is necessary to produce a key within a short period. Our proposed method takes only a small period of key generation time compared to other methods.

**Fig. 8:**
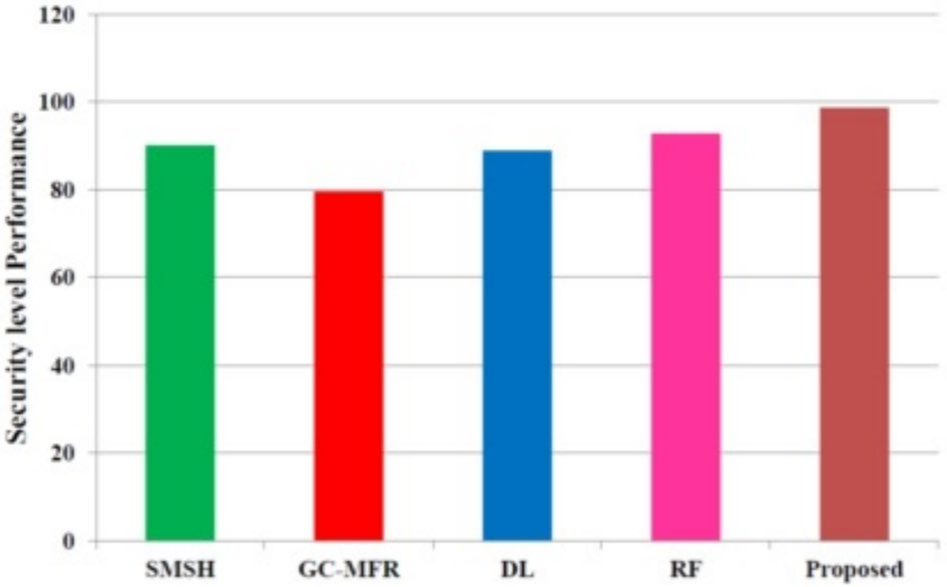
Performance evaluation based on the security protection.

Figure 8 expresses the performance evaluation based on security protection. Security is an important aspect of any kind of data storage. In our approach, the IoT-based system ensures better security by about 98 %. The inclusion of the matrix-based RSA approach enhances the security protection and other approaches such as SMSH, GC-MFR, DL, and RF ensure security levels of 90%, 89%, 91%, and 93% respectively.

**Fig. 9:**
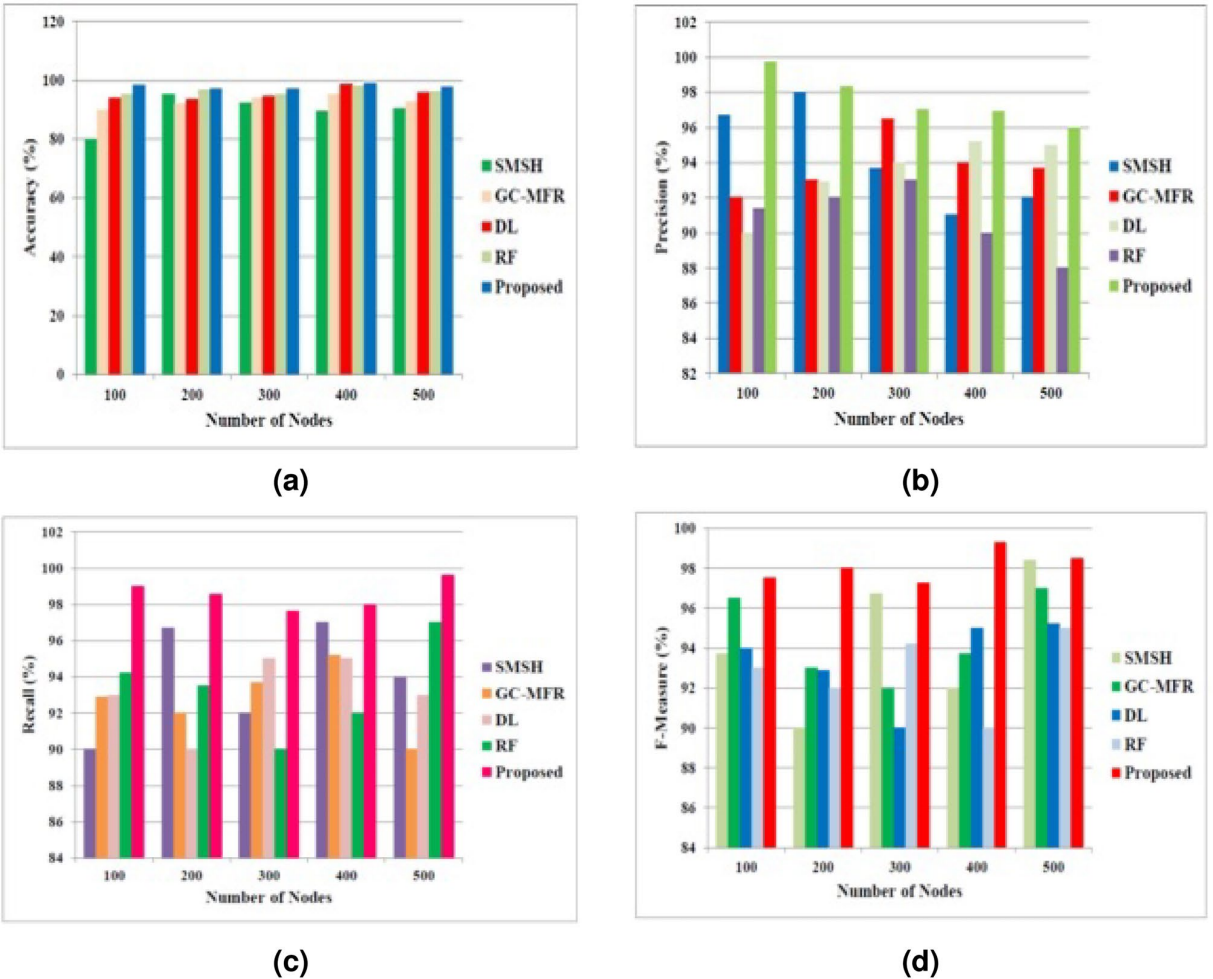
Performance evaluation based on the (**a**) Accuracy, (**b**) Precision, (**c**) Recall, and (**d**) F-measure.

The performance based on the ML parameters are illustrated in Fig. 9. Our proposed approach uses the DCNN-based AO technique to classify heart disease better from the accessed documents. This method produces a better global search and thus ensures the accurate heart disease prediction from the given dataset. Figure 9a shows that the proposed method has higher accuracy than the other approaches. It also ensures better precision, recall, and F1-measure. The predicted precision, recall, and F1-measure values are shown in Fig. 9b–d correspondingly. From all graphical representations, it is noted that our proposed approach has better values than the other methods.

Encryption and decryption times are the primary metrics used to validate the suggested model’s latency. It takes time to encrypt data from plaintext to ciphertext and time to decrypt data from ciphertext back to plaintext. The suggested model’s expected encryption and decryption times are displayed in Table 9, and as per the results, the proposed model finishes the operation rapidly to maintain the real-time standards. The latency, aka execution time, is computed using the average encryption and decryption times. The computations are conducted by varying the input sizes with the same key.

We evaluate the suggested model against state-of-the-art methods using f-measure, accuracy, and precision, and we compare it to RF^18^, DL^43^, ANN^44^, and the Firefly algorithm^45^. Table 10 displays the outcomes of the tests performed in the testing dataset that was constructed using the UCI heart disease dataset. Using the AO algorithm to fine-tune the CNN architecture’s hyperparameters yields better performance in comparison to state-of-the-art methods, as demonstrated by the results.

**Table 9:** Encryption and decryption times for proposed matrixbased RSA algorithms.

Size of input text (bytes)	RSA algorithms used	Average encryption times (ms)	Average decryption times (ms)
16	Existing RSA	0.1548	0.1645
	Matrix-based RSA encryption method	0.1165	0.1245
32	Existing RSA	0.2854	0.2954
	Matrix-based RSA encryption method	0.2154	0.2245
64	Existing RSA	0.3965	0.4015
	Matrix-based RSA encryption method	0.3214	0.3325
128	Existing RSA	0.5487	0.5547
	Matrix-based RSA encryption method	0.5326	0.5478

**Table 10:** Comparative analysis using the UCI heart disease dataset.

Technique	Accuracy	F-Score	Precision
RF^18^	0.9145	0.9275	0.9117
DL^43^	0.9245	0.9241	0.9365
ANN^44^	0.9314	0.9297	0.9452
Firefly algorithm^45^	0.9254	0.9147	0.9014
Proposed	0.9857	0.9857	0.9854

**Fig. 10:**
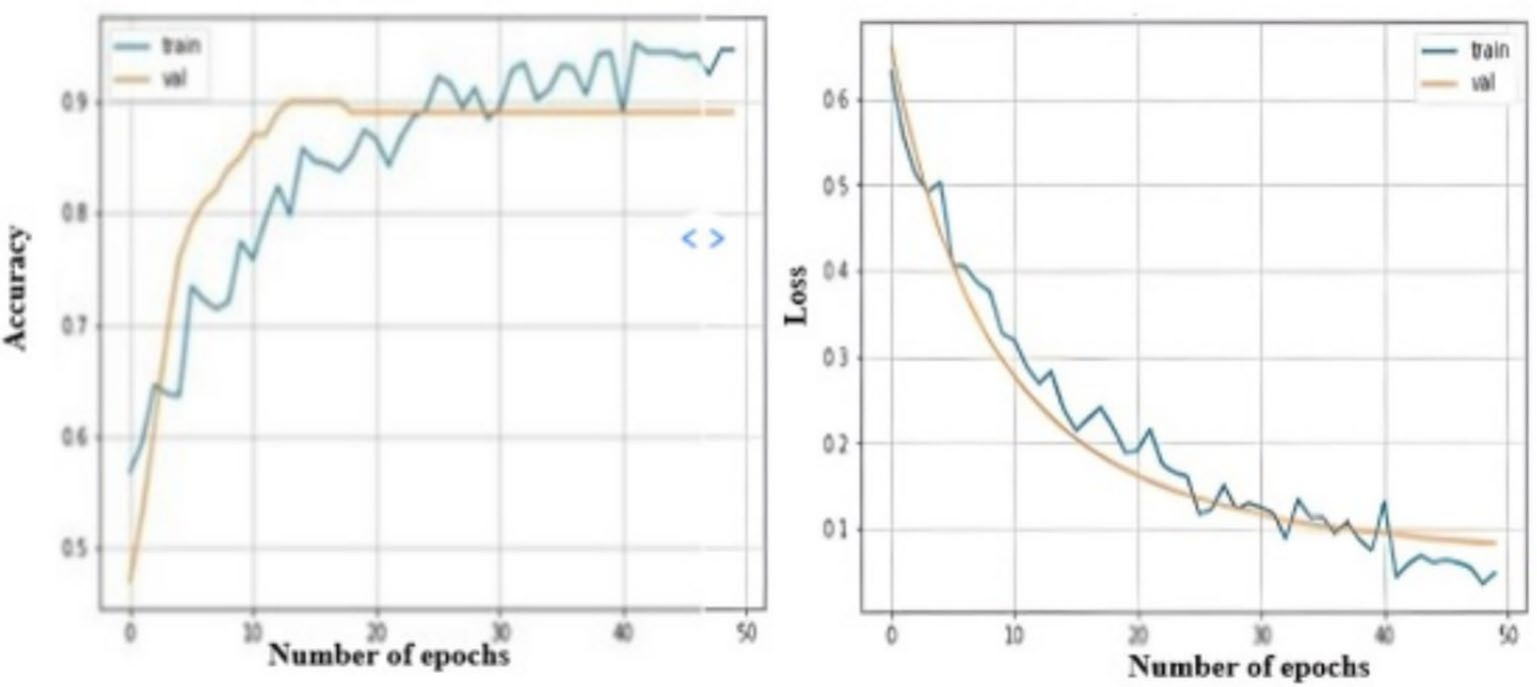
Accuracy and loss curve of the proposed model.

To better understand the learning performance fluctuations over time and to identify any learning issues that could lead to an underfit or overfit model, the training and validation loss values are useful. They will also supply the epoch for the inferencing step to utilize in conjunction with the training model’s weights. Figure 10 displays the suggested model’s accuracy and loss curves. The performance of a classification system can be illustrated using a confusion matrix, which is a table. A confusion matrix is a graphical representation and summary of a classification algorithm’s output.

**Fig. 11:**
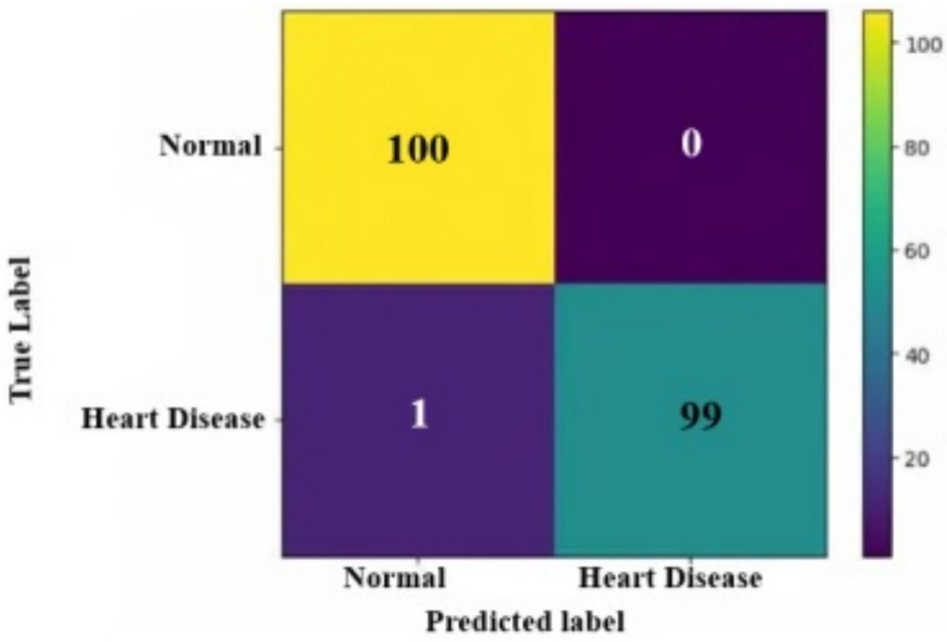
Confusion matrix for heart disease prediction.

**Fig. 12:**
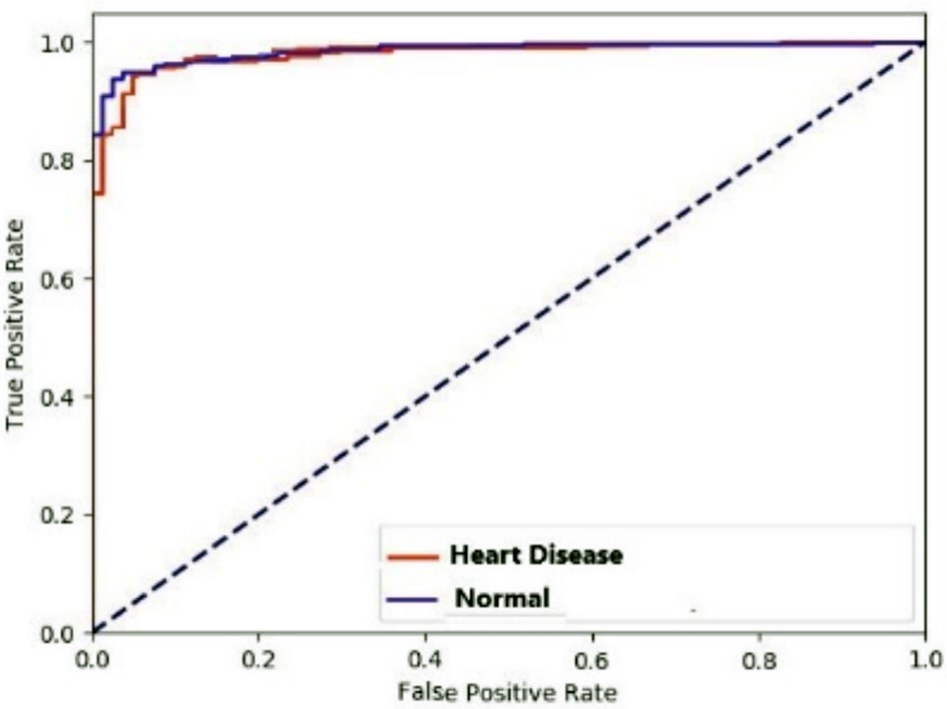
ROC curve for heart disease prediction.

A table called a confusion matrix is used to describe how well a classification system performs. The output of a classification algorithm is visualized and summarised in a confusion matrix. Figure 11 displays a confusion matrix where normal input is referred to as normal, and inputs with deviated conditions are seen as heart disease. When the ROC curve is shaped like a right angle, it produces false positives as often as it does real positives. Due to this, a diagnostic test ought to feature a ROC curve situated in the top left triangle, over the y=x line. Whereas a perfect discriminating capacity is shown by an AUC of 1.0, a test with no discriminating ability (i.e., no better than chance) is denoted by an AUC of 0.5. As a broad indicator of a test’s discriminatory power, the area under the receiver operating characteristic (ROC) curve (AUC) helps determine if cardiac illness is present. The ROC curve for the normal and heart disease datasets is presented in Fig. 12. The model was trained with stratified sampling to ensure a balanced representation across different temporal spans, and performance metrics were consistently high across both complete and partial data subsets. Furthermore, the architecture includes temporal abstraction mechanisms within the convolutional layers, enabling it to capture relevant patterns even from shorter monitoring windows. Empirical validation showed minimal deviation in accuracy and F1-score between full and partial sequences, demonstrating the model’s resilience.

By incorporating a multi-input DCNN architecture that can handle diverse biomedical signals like ECG, EEG, SpO$_2$ , glucose levels, and body temperature, the suggested IoT-SHMF framework can be technically enhanced to enable real-time monitoring and prediction for numerous diseases. Regarding disease-specific feature extraction layers, each input modality goes via parallel convolutional pathways. This allows for both shared and differentiated pattern recognition. The Archimedes Optimisation Algorithm makes dynamic adjustments to convolutional filter sizes, learning rates, and dropout rates for each disease-specific branch to guarantee optimum performance and convergence across different clinical datasets. Secure transfer of patient data from several sources to an inference engine, whether central or distributed, is achieved by matrix-based RSA encryption. A multi-label output node system is used to reorganize the classification layer, allowing for the simultaneous and highly accurate identification of various illness classifications. Assuring low-latency computing and retaining diagnostic accuracy across cardiovascular, respiratory, metabolic, and neurological illness profiles, real-time inference is made possible by the edge-device deployment of quantized and pruned model variations.

## Conclusion

This study presented a novel IoT-enabled and secured healthcare monitoring framework for heart disease prediction. JAVA software handles the implementation of the proposed work. The data are gathered from different medical sensor devices and are divided into normal and abnormal. The training and testing accuracy of the proposed method is higher, and the loss function is lower. The precision, recall, security analysis, F-measures, Encryption time, and decryption times are used to validate the performance of the proposed method. The proposed techniques’ encryption and decryption times are lower than those of other existing techniques. The proposed IoT-based system ensures better security by about 98%. When there are 25 sensor nodes, our proposed approach has a decryption time of 37 seconds. The proposed method takes only a small period of key generation time compared to other methods. In the future, we hope to create a lightweight disease prediction model for COVID-19 that can adapt to dynamic changes in the environment, minimize latency, and eliminate fraudulent blockchain transactions in the devices of edge users. Advanced encryption techniques will also improve the developed model’s privacy. The study does, however, have significant flaws. Some examples in the dataset utilized in this study do not have a five-week learning period, resulting in inadequate valid data for the experiment. The proposed model will be extended to identify COVID-19, pneumonia, chikungunya, etc. We will improve the performance by increasing the resource diversity and number of features. We also plan to choose an optimal feature subset via a novel feature extraction technique that enhances the performance. Research into intelligent healthcare systems and medical diagnostics stands to benefit greatly from the planned IoT-SHMF using DCNN-AO. First, it shows how bio-inspired optimization methods, combined with deep learning, improve computing efficiency and model accuracy in real-time health monitoring. This integration has created new opportunities for developing self-optimizing, adaptive diagnostic systems that can reliably function in IoT situations with limited resources. Second, to tackle the crucial problem of data privacy and trust in healthcare IoT networks, the framework stresses the increasing significance of incorporating strong security measures into predictive models. One key limitation is the computational complexity of the Deep Convolutional Neural Network, which may hinder real-time processing on resource-constrained IoT edge devices. Additionally, the model’s performance depends on the training data’s quality, volume, and diversity; insufficient or imbalanced datasets can lead to biased predictions or reduced accuracy^46–50^.

## Data Availability

Data used in this study are publicly available and were obtained from the UCI Machine Learning Repository - Heart Disease dataset (https://archive.ics.uci.edu/ml/datasets/heart+disease).
